# Analysis of Hindgut Microbiome of Sheep and Effect of Different Husbandry Conditions

**DOI:** 10.3390/ani11010004

**Published:** 2020-12-22

**Authors:** Giulietta Minozzi, Filippo Biscarini, Emanuela Dalla Costa, Matteo Chincarini, Nicola Ferri, Clara Palestrini, Michela Minero, Silvia Mazzola, Renata Piccinini, Giorgio Vignola, Simona Cannas

**Affiliations:** 1 Dipartimento di Medicina Veterinaria, Università degli Studi di Milano, 20133 Milan, Italy; emanuela.dallacosta@unimi.it (E.D.C.); clara.palestrini@unimi.it (C.P.); michela.minero@unimi.it (M.M.); silvia.mazzola@unimi.it (S.M.); renata.piccinini@unimi.it (R.P.); simona.cannas@unimi.it (S.C.); 2 CNR-IBBA, CNR, 20133 Milan, Italy; biscarini@ibba.cnr.it; 3 Facoltà di Medicina Veterinaria, Università degli Studi di Teramo, 64100 Teramo, Italy; mchincarini@unite.it (M.C.); gvignola@unite.it (G.V.); 4 Istituto Zooprofilattico Sperimentale dell’Abruzzo e del Molise G. Caporale, Campo Boario, 64100 Teramo, Italy; n.ferri@izs.it

**Keywords:** sheep, hindgut microbiome, husbandry conditions, physiology, hair cortisol

## Abstract

**Simple Summary:**

The purpose of this study was to describe the composition of the hindgut microbiome in sheep and to test whether different husbandry conditions could have an effect in changing the composition and the diversity of the hindgut microbiome, based on the assumption that there is a known influence between stress-related husbandry conditions and the gut microbiome. The results of our study demonstrated very few differences in the sheep hindgut microbiome, basically related to *Verrucomicrobia* abundance, when compared with previous studies. Additionally, the investigation of the interactions between microbiome and animal husbandry showed few indicators of difference between groups, which might indicate the presence of a low-level stress across the flock, depending on management procedures. Surely, this work represents a contribution for the analysis of the microbiome in animal production and welfare research.

**Abstract:**

The microbiome is now seen as an important resource to understand animal health and welfare in many species. However, there are few studies aiming at identifying the association between fecal microbiome composition and husbandry conditions in sheep. A wide range of stressors associated with management and housing of animals increases the hypothalamic–pituitary axis activity, with growing evidence that the microbiome composition can be modified. Therefore, the purpose of the present study was to describe the core microbiome in sheep, characterized using 16S rRNA gene sequencing, and to explore whether exposure to stressful husbandry conditions changed sheep hindgut microbiome composition. Sheep (*n* = 10) were divided in two groups: isolated group (individually separated for 3 h/day) and control group (housed in the home pen for the entire trial period). Sheep core microbiome was dominated by Firmicutes (43.6%), Bacteroidetes (30.38%), Proteobacteria (10.14%), and *Verrucomicrobia* (7.55%). Comparative results revealed few operational taxonomic units (OTUs) with significantly different relative abundance between groups. Chao1, abundance-based coverage estimator (ACE), and Fisher’s alpha indices did not show differences between groups. OTU-based Bray–Curtis distances between groups were not significant (*p*-value = 0.07). In conclusion, these results describing the core microbiome of sheep do not suggest a strong effect of stressful husbandry conditions on microbial composition.

## 1. Introduction

The intestinal microbiome includes microorganisms fundamental for host physiology, immunity, and the central nervous system in both humans and animals [1,2,3]. Today, this omics approach has been largely used in livestock [4,5,6]. However, there are some very recent results on the characterization of the hindgut microbiome in sheep; among them are the study by Tanca A. et al. that characterized the microbial composition of different animals, including the sheep [7], and two papers that report the result of research based on a multi-omics approach to investigate the fecal proteome of a local sheep breed [8,9]. More recently, a study conducted in merino sheep analyzed the composition and the stability of the fecal microbiota. The authors demonstrated high short-term stability of the microbiota in this species and identified that Firmicutes and Bacteroidetes were the predominant bacterial phyla, constituting almost 80% of the total population [10]. In addition, recent studies aimed to define a core gut microbiome across sheep breeds have analyzed its composition in different breed differences as the case of the study on the Tibetan sheep breed [11]. Finally, other studies have tested and verified the influence of supplements or different feeding regimens in altering the composition of the sheep gut microbiome [12]. Moreover, there is growing evidence that the microbiome composition can be modified by stressors, such as maternal separation, physical restraint, and overcrowding [13,14]. The hindgut microbiome composition has been demonstrated to be altered in social defeat stress in mice. Bharwani et al. [15] found a reduced diversity associated with a decrease in the abundance of Clostridium species, while an opposite result was found by Bailey et al. [16]. Finally, despite the opposite results, both these animal studies demonstrated an association between the altered intestinal microbiome and depressive-like behavior [17]. The concept that different husbandry conditions, mainly related to management practices and housing, can affect animal welfare is well known in livestock science and animal husbandry [18,19,20,21]. For instance, it has been proven that negative emotional experiences induce chronic mild stress in sheep [22,23]. One of the most important features of the behavior of sheep is their marked sociality; sheep show a strong need to stay with their group, and when isolated from their flock mates, they show behavioral and physiological responses indicative of stress [24,25,26,27,28,29,30]. Therefore, it is interesting to study the interplay between husbandry conditions, animal welfare, and the hindgut microbiome in sheep. The present study aimed to: (i) characterize the hindgut microbiome of sheep, using next generation sequencing (NGS) of the V3–V4 hypervariable regions of the 16S rRNA and (ii) explore whether exposure of adult sheep to stressful husbandry conditions (isolation vs. control) significantly changed the fecal microbiome composition. The effect of husbandry condition is investigable through cortisol titrations in hair [31]. Accordingly, the relationship between changes in the hindgut microbiome and hair cortisol concentrations was evaluated.

## 2. Materials and Methods

### 2.1. Animals and Housing

Ten non-lactating nor gestating Sarda breed ewes, aged 6 months, were selected for this study. All the sheep originated from the same herd and, after 20 days of adaptation, were assigned into two groups balanced by weight (25.11 ± 2.60): isolated (*n* = 5) and control (*n*= 5). Each group was housed in a home pen (20 m2) at the “Istituto Zooprofilattico Sperimentale dell’Abruzzo e del Molise” (Italy). Diet was composed of first cut alfalfa (estimated daily consumption 600 g DM/head), supplemented with a commercial concentrate pellet (Mangimi Ariston Srl, Teramo, Italy; 250 g/head/d). All animals had free access to water.

### 2.2. Experimental Plan

The experiment started in April 2018 and lasted 8 weeks. The sheep of the isolated group were individually separated for 3 h/day. The individual pen (length: 2 m; width: 0.80 m) was designed to allow individual isolation without visual and tactile contact. Sheep were isolated in a different pen each day to avoid habituation. The control group was housed in their home pen for the entire trial period. Many studies in sheep utilize isolation as a source of stress; the duration of isolation varied from 5 min to 24 h [26,32,33]. Repetitive isolations in blocks of 3 h were used to study the stress response in sheep [34], and even repetitive isolation led to a persistent and reproducible stress reaction with only little habituation over time [35].

### 2.3. Hair Collection

Hair was collected from the right shoulder region of each sheep, which was clipped to the skin on the first day of the experiment [31]. On day 57 (the last day of the experiment, exactly the same day as when the fecal sample was collected), a sufficient amount of actively growing hairs was collected in order to provide reliable results [36]. The hair samples were stored in tin foil bags at room temperature until analysis.

### 2.4. Cortisol Measurement

Extraction of hair cortisol was performed following the procedure described by Burnett et al. [37] with some modifications. One hundred mg of each sample of clean and dried ovine hair was weighed and placed in a vial. Then, 2 mL of 99.9% methanol (Sigma-Aldrich, Milano, Italy) was added. The vials were tightly capped and sonicated for 30 min. The samples were then incubated overnight at 100 rpm and 50 °C to extract the steroids, and then 1.5 mL of the original volume of methanol was pipetted into a 2.5 micro-centrifuge tube and evaporated at 45 °C under a stream of ultrapure nitrogen gas. The samples were reconstituted in 200 μL of phosphate buffered saline (PBS) (Merk Millipore, Milano, Italy). Hair cortisol was analyzed using a commercially available assay kit designed for accurately measure cortisol levels in a variety of sample matrices (Enzo Life Sciences, Farmingdale, New York, NY, USA). Samples were aliquoted into wells in duplicate (100 μL), and absorbance was measured using a wavelength of 405 nm in a microplate plate reader (Multiskan EX, LabSystem, Thermo Fisher Scientific, Milan, Italy).

### 2.5. Sampling of the Hindgut Microbiome

After 8 weeks of treatment (isolation and control), fecal material was collected directly from the rectal ampulla of each animal after accurate cleaning of the anal region. The samples were immediately frozen at −20 °C. Overall, 10 samples were subjected to high throughput sequencing. This study has been approved by the Italian national ethical commission (Italian Ministry of health authorization n° 457/2016-PR).

### 2.6. 16 S rRNA-Gene Sequencing

DNA was extracted from each fecal sample using a QIAmp DNA Stool kit (Qiagen, Hilden, Germany), according to the manufacturer’s protocol. DNA quality and quantity were assessed using a Nano Drop ND-1000 spectrophotometer (Nano Drop Technologies, Wilmington, DE, USA). The isolated DNA was then stored at −20 °C until use. Bacterial DNA was amplified using the primers described in literature [38], which target the V3–V4 hypervariable regions of the 16S rRNA gene. For each reaction, 2 μL of genomic DNA (5 ng/μL), 0.2 μL of each primer (100 μM), and12.5 μL of KAPA HIFI Master Mix 2 × (Kapa Biosystems, Inc., MA, USA) were used; the specific buffer was added to reach a final volume of 25 μL/sample. Blank controls (no DNA template added to the reaction) were also run in each PCR. A first amplification step was performed in an Applied Biosystem 2700 thermal cycler (Thermo Fisher Scientific). Samples were denatured at 95 °C for 3 min, followed by 25 cycles with a denaturing step at 98 °C for 30 s, annealing at 56 °C for 1 min, and extension at 72 °C for 1 min, with a final extension at 72 °C for 7 min. Amplicons were cleaned with Agencourt AMPure XP (Beckman, Coulter Brea, CA, USA), and libraries were prepared following the 16S Metagenomic Sequencing Library Preparation Protocol (Illumina, San Diego, CA, USA). The libraries obtained were quantified by Real Time PCR with KAPA Library Quantification Kits (Kapa Biosystems, Inc., MA, USA), pooled in equimolar proportion, and sequenced in one MiSeq (Illumina) run with 2 × 250-base paired-end reads. The 16S rRNA gene sequences obtained in this study were deposited in the EMBL-EBI European Nucleotide Archive (ENA) database (study ID PRJEB31150).

### 2.7. Bioinformatics Processing

Demultiplexed paired-end reads from 16S rRNA-gene sequencing were first checked for quality using FastQC [39] for an initial assessment. Forward and reverse paired-end reads were joined into single reads using the C++ program SeqPrep [40]. After joining, reads were filtered for quality based on: (i) maximum three consecutive low-quality base calls (Phred < 19) allowed; (ii) fraction of consecutive high-quality base calls (Phred > 19) in a read over total read length ≥ 0.75; (iii) no “*n*”-labeled bases (missing/uncalled) allowed. Reads that did not match all the above criteria were filtered out. All remaining reads were combined in a single FASTA file for identification and quantification of OTUs (operational taxonomic units). Reads were aligned against the SILVA closed reference sequence collection release 123, with 97% cluster identity [41,42], applying the CD-HIT clustering algorithm [43]. A pre-defined taxonomy map of reference sequences to taxonomies was then used for taxonomic identification along the main taxa ranks down to the genus level (domain, phylum, class, order, family, genus). By counting the abundance of each OTU, the OTU table was created and then grouped at each phylogenetic level. OTUs with total counts lower than 10 in fewer than 2 samples were filtered out. All of the above steps, except the FastQC reads quality check, were performed with the QIIME open-source bioinformatics pipeline for microbiome analysis [44].

### 2.8. Alpha and Beta Diversity Indices

Analyses were done on the entire group of 10 animals to describe the hindgut sheep microbiome as a whole, and then within the two different groups (isolated and control) to compare the two husbandry conditions. Additionally, pairwise comparisons between groups were conducted. Differences with *p* < 0.05 were considered significant. The Firmicutes to Bacteroides (F/B) ratio in the two experimental groups was also estimated.

To evaluate the phylogenetic composition of the bacterial communities in the sheep hindgut samples, we first looked at taxonomies from OTU counts and then used different indices to estimate within- and among-sample variability. Within-sample microbial richness, diversity, and evenness were calculated by the following indices: Chao1 and ACE (abundance-based coverage estimator) for richness [45,46,47]; Shannon, Simpson, and Fisher’s alpha for diversity; equitability (Shannon evenness) and Simpson E for evenness [48,49,50]. Across-sample hindgut microbiome diversity was estimated calculating Bray–Curtis dissimilarities [51]; among groups (isolated, control) and pairwise, dissimilarities were evaluated non-parametrically using the permutational analysis of variance approach. Calculations of all mentioned indices are described in Biscarini et al. [4].

### 2.9. Statistical Analysis

Differences in cortisol concentration between isolated and control group were assessed by Mann–Whitney test. All statistical analysis and graphical representations were produced using the R environment for statistical programming.

The comparison between groups to determine differences in alpha diversity indices was carried out by one-way analysis of variance (ANOVA) with a cutoff value of 0.05 (*p* < 0.05) for significant difference. To determine differences in taxa abundance between groups, a differential relative abundance analysis (DAA) based on one-way ANOVA was conducted, and the same *p* < 0.05 cut-off value was used to determine significant differences.

The between-group differences in terms of alpha diversity indices and taxa abundances were also evaluated with a Bayesian approach. The data were considered to be independent and identically distributed (i.i.d.) draws from *t* distributions with different means (mu) and standard deviations (sigma) and a common normality parameter nu (that controls the thickness of the tails). Minimally informative priors were chosen: normal priors with large standard deviation for (μ), broad uniform priors for (σ), and a shifted-exponential prior for (ν) [52]. From the posterior distribution of the parameters conditional on the data, the distribution of differences between average values in the two groups was obtained. We set a threshold corresponding to 10% of the mean of the quantity of interest across all samples (e.g., average value of any alpha diversity index or average counts for any specific taxon) and estimated the probability for the difference between the two groups (μ1–μ2) to be larger than this threshold. The R package *BEST* was used to implement the described Bayesian estimation approach [53].

Bray–Curtis dissimilarities were evaluated non-parametrically via permutational analysis of variance (PERMANOVA) by using 1000 permutations repeated 500 times.

## 3. Results

### 3.1. Hair Cortisol Concentration

At the end of the follow-up period, isolated sheep and control ones did not show significant differences in hair cortisol concentrations (mean values 0.91 ng/mg vs. 0.86 ng/mg).

### 3.2. Sequencing Results and Taxonomy Description

A total of 10 hindgut samples were analyzed. Sequencing the V3–V4 regions of the bacterial 16S rRNA gene produced a total of 2,736,335 reads (joined R1–R2 paired-end reads), with an average of 273,633 reads per sample. After quality filtering, 578,326 sequences were removed, leaving 2,158,009 sequences for subsequent analyses (78.7% average retention rate, maximum 81%, minimum 76%).

The initial number of OTUs identified was 9134; after pruning out OTUs with fewer than 10 counts in at least two samples, 2864 distinct OTUs were left. To check whether sequencing depth and sample size were adequate to characterize the composition of the sheep hindgut microbiome, sequence-based and sample-based rarefaction curves were generated from the OTU table before pruning (9134 OTUs). Sequence-based rarefaction curves were obtained from the QIIME pipeline (r-project.org). The sample-based rarefaction curve was produced with ad hoc R functions. The observed number of OTUs detected was plotted as a function of the number of reads in each sample and of the number of samples. Both curves tended to plateau asymptotically towards a maximum, indicating that sequencing depth and the number of samples were adequate to characterize the sheep hindgut microbiome in the present study (data not shown).

The results of the NGS analysis of the 16S rRNA gene showed that the microbiome was dominated by OTUs/microbial species belonging to few main taxonomic phyla: Firmicutes that accounted for 43.6% of the hindgut microbiome, followed by Bacteroidetes (30.38%), Proteobacteria (10.14%), and *Verrucomicrobia* (7.55%). Altogether, the remaining 13 phyla detected made up for 8.3% of the hindgut microbiome. A detailed composition at phylum level is given in Table 1. Down the phylogenetic classification, the most representative taxa were the classes Clostridia (41.5%) and Bacteroidia (30.1%), the orders Clostridiales (41.5%) and Bacteroidales (30.16%), the families *Ruminococcaceae* (28.6%), Rikenellaceae (10.5%), and Campylobacteraceae (7.9%), and the genera *Ruminococcaceae* UCG-010 (8.8%) and Campylobacter (7.9%) (Table 2).

In order to identify specific bacterial species related to the different husbandry conditions, we explored the taxonomy of the most differentially abundant taxa in each group (Table 3).

The phylum Fibrobacteres was the only differentially expressed one (*p* = 0.0293). Further down the phylogenetic classification, the most differentially represented were the Fibrobacteria (*p* = 0.0293), the orders Aeromonadales (*p* = 0.01), Desulfovibrionales (*p* = 0.004), Fibrobacterales (*p* = 0.029), Micrococcales (*p* = 0.029), and Thermoanaerobacteriales (*p* = 0.013), the families Defluviitaleaceae (*p* = 0.01), Desulfovibrionaceae (*p* = 0.004), and Fibrobacteraceae (*p* = 0.02), and the genera *Ruminococcaceae* UCG-011 (*p* = 0.002), Lachnoclostridium 10 (*p* = 0.004), and Fibrobacter (*p* = 0.029). Results from the Bayesian analysis confirmed all the significant differences from Table 3, with probabilities for the difference to be larger than the threshold in the range 0.88–0.98.

**Figure 1:**
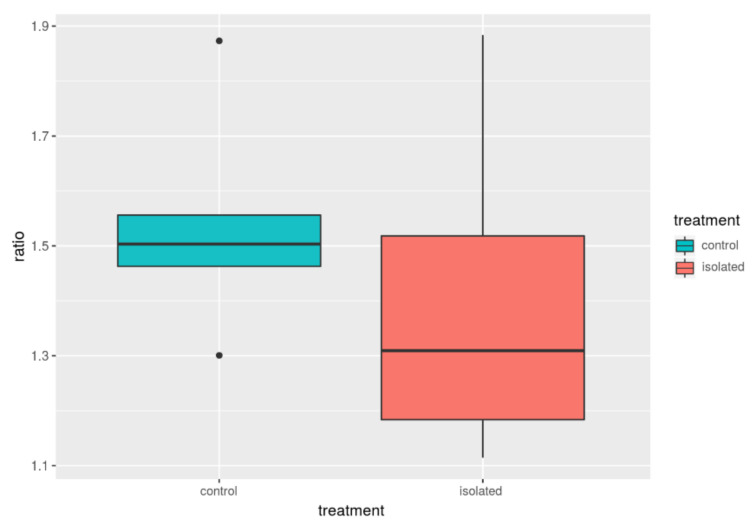
F/B ratio between control and isolated animals.

The mean F/B ratio was estimated, and the results were 1.40 and 1.54 for isolated and control groups, respectively; however, the difference was not statistically significant (*p*-value = 0.436; Table 4 and Figure 1). Further analysis of the F/B ratios based on a Bayesian reformulation of the problem estimated an average difference of 0.137 from the posterior distribution of the parameters and a probability of 0.72 for this difference being greater than zero. However, the 95% credibility interval for the between-group difference included the 0 [−0.47, 0.73].

### 3.3. Diversity Indices

The estimated alpha diversity indices for richness, diversity, and evenness of the hindgut microbiome of the 10 sheep are reported in Table 5 along with standard deviations.

Overall, the mean number of observed OTUs was 2592.7, and the average Chao1 and ACE values were 2907.8 and 2902.9, respectively. Regarding the difference among the two experimental groups, the average number of observed OTUs was similar, as was the diversity index Fisher’s and alfa richness estimators Chao1 and ACE (Table 6). None of these differences were significant from the analysis of variance; the Bayesian model confirmed that the probabilities that these between-group differences were larger than the margin thresholds were all very low (between 0.001 and 0.131, except for Simpson_e, where it was 0.776).

**Figure 2:**
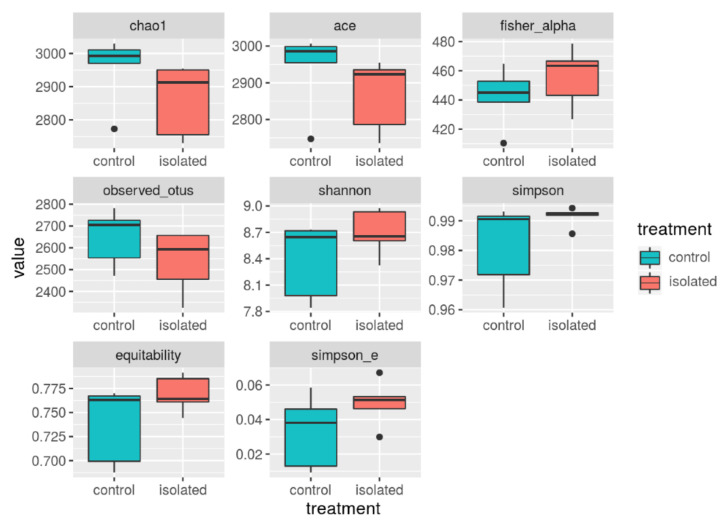
Estimated alpha diversity indices in the sheep hindgut microbiome from the two treatment groups.

However, as shown in Figure 2, the samples showed high variability.

**Figure 3:**
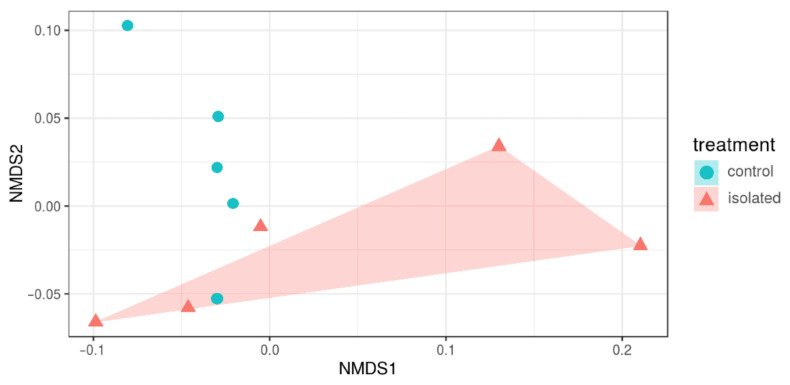
Bray–Curtis dissimilarity matrix between the 10 sheep handled under different husbandry conditions. First two dimensions from the (non-metric) multi-dimensional scaling (NMSD1 and NMSD2) of the Bray–Curtis dissimilarity matrix. Samples were grouped by experimental unit (treatment: isolated; control).

Bray–Curtis clustering of the fecal bacteria at the OTU level showed that the samples of the two experimental groups did not cluster separately (Figure 3), and the distances were not significantly different between treatments, with an overall *p*-value = 0.073. The permutational analysis of variance (PERMANOVA), with 999 permutations repeated 500 times, suggested that the microbial community structure of the animals that underwent stressful husbandry conditions did not create an overall differential composition of the microbiome to be identified, even though some clustering tendency could be seen from the first two principal dimensions.

## 4. Discussion

The purpose of this study was, on the one hand, to describe the composition of the hindgut microbiome in sheep, and, on the other hand, to test whether different husbandry conditions could have an effect in changing the composition and the diversity of the hindgut microbiome based on the assumption that there is a known and reciprocal influence between stress-related husbandry conditions and the gut microbiome, probably through the gut–brain axis [54,55]. In order to check the level of stress in the isolated sheep compared to the control, we tested the hair cortisol levels at the end of the follow-up period. Measurement of cortisol is an indicator for the hypothalamic–pituitary–adrenocortical axis (HPA) activity, which reflects the physiological responses to acute or long-term stress [56,57]. The common biological matrices for the analysis of cortisol are serum, saliva, urine, and feces, but in these substrates, the cortisol levels represent a short retrospective timespan (few minutes up to 1–2 days) [58]. Conversely, the hair cortisol concentration is a marker of cortisol secretion and stress over long periods of time [59]. Its quantification is increasingly used in psycho-neuro-endocrinological studies in humans, and, more recently, also in animal stress and welfare research, including sheep [31,58,59,60,61]. The sampling procedure, smooth and minimally invasive, and the extended time periods in which the data were obtainable from a sample refer make the hair cortisol titration an extremely useful biomarker for the assessment of chronical stress in animals, although, to date, no species-specific reference values are available. The lack of significant differences between groups could be interpreted in the light of the demonstrated attitude of some sheep to be low-responder, with a low HPA reactivity to managerial stress [62]. Actually, we found minor differences in the microbiome of experimental and control animals. In accordance with previous studies [7,8], the most and the second-most abundant phylum were, overall, Firmicutes and Bacteroidetes (43.6% and 30%, respectively), followed by Proteobacteria and *Verrucomicrobia*. Additionally, a recent study on merino sheep [10] identified the two phyla as dominating the microbiome, which indicates a stability of the core microbiome across breeds. Differently from the above cited studies, we found a markedly lower abundance of *Verrucomicrobia*. Going down the phylogenetic classification, Mamun et al. identified the *Ruminococcaceae* and an unassigned family belonging to order Bacteroidales as the most abundant families. Our data are almost identical if we classify the unassigned family as Rikenellaceae. The results of the present study showed that the isolated sheep had the same microbiome richness and variability as the control group; indeed, Chao1 and ACE indices did not show a statistical difference. To the contrary, a recent study regarding human depressive statuses found an association with lower gut microbiome richness in major depressed patients in comparison with healthy controls [63]. It must be noticed that, in the same study, the authors found no significant differences in the Shannon diversity indices nor in the Firmicutes to Bacteroidetes ratio, even if the F/B ratio is considered an indicator of dysbiosis of the human gut microbiome [64]. This picture is consistent with our findings. Differently, recent research addressing the microbiome composition of the calf in relation to the stress caused by dehorning or castration has shown that the F/B ratio was significantly reduced in the animals undergoing a higher stress level [55]. In this case, the discordance of our results could be due to the lack of pain and lower intensity of stress imposed, which was confirmed by the similar levels of hair cortisol in the two groups. Due to the small sample size, we cannot conclude definitively that there is no difference in the F/B ratio; actually, we observed a sizable difference (1.54–1.40), but the sample size was too small to reveal significance in the classical sense. However, both the Bayesian and the non-parametric bootstrapping (results not shown) analyses give credit to the existence of an actual difference (although, again, not conclusively).

When compared with a previous paper regarding the ruminant hindgut microbiome [7], our data showed a similar mean abundance of Proteobacteria in both groups (0.120 and 0.082 in treated and control group, respectively, vs. 1.68).

The most salient differences between groups were observed in hindgut microbiome composition: the phylum Fibrobacteres, including its order, family, and genus, showed higher abundance in the control group compared to the isolated animals, as did Aeromonadales, Micrococcales, Desulfovibrionales, and Thermoanaerobacterales. A previous study on dairy cows and sheep reported that cellulolytic bacteria (Fibrobacteres) were reduced in the presence of sub-acute ruminal acidosis [65]. In our experiment, diet was the same in both groups, and no acute stress that could cause acidosis characterized the treated group according to cortisol data. Therefore, our results need to be further investigated in order to understand the potential role of a low and chronic stress factor on light alterations of sheep hindgut microbiota.

Interestingly, as in the study conducted on mouflon and blue sheep kept at high altitude compared to low altitude [66], we found higher Firmicutes/Bacteroidetes ratio and higher *Ruminococcaceae* in the control group as in the high altitude group. These results indicate a possible beneficial effect for the sheep in producing gut microbiota-mediated energy and a better ability in starch decomposition in these conditions [66].

Bray–Curtis distances showed that the two groups were not significantly separated. This result could be explained by the low HPA reactivity, which minimizes the major consequences of stress. The daily but timely short isolation of the group that was regarded as a stress factor probably did not represent a sufficient stressor to cause significant differences in the microbiome composition. On the other hand, our findings might indicate that control animals shared the same level of stress as isolated animals. Indeed, the interaction between the animal and the stockman with repeated handling and moving, the milking management, and the changes in social conditions are potential stress factors for housed sheep [67]. This leads to considering encouragement to better adapt the farm environment to the sheep by improving management practices and housing conditions, also providing higher environment enrichment. Indeed, the effects of environmental enrichment elements have been shown to enhance pig and lamb welfare, synaptic plasticity, learning performance, and memory [18,19,20]. These enrichment elements should be considered to increase the wellness and the welfare of animals.

## 5. Conclusions

In spite of the limitation of the experiment due to the small number of animals, the results of the present study demonstrated very few differences in the sheep hindgut microbiome, basically related to *Verrucomicrobia* abundance, when compared with previous studies. Additionally, the investigation of the interactions between microbiome and animal husbandry showed few indicators of difference between groups, which might indicate the presence of a low-level stress across the flock, depending on management procedures. Surely, this work represents a contribution for the analysis of the microbiome in animal production and welfare research.

## Figures and Tables

**Table 1:** Composition at phylum level of the sheep hindgut microbiome in the two husbandry conditions (control and isolated) and the *p*-value of their relative difference (relative abundances).

Phylum	Control	Isolated	*p*-Value
Firmicutes	0.4232	0.4496	0.077
Bacteroidetes	0.2782	0.3294	0.246
Proteobacteria	0.1204	0.0824	0.110
*Verrucomicrobia*	0.0870	0.0639	0.233
Cyanobacteria	0.0151	0.0134	0.116
Fibrobacteres	0.0210	0.0058	0.029
Lentisphaerae	0.0130	0.0106	0.068
Spirochaetae	0.0118	0.0102	0.213
Euryarchaeota	0.0100	0.0103	0.137
Tenericutes	0.0090	0.0101	0.257
Planctomycetes	0.0044	0.0033	0.089
Saccharibacteria	0.0019	0.0036	0.528
Actinobacteria	0.0025	0.0027	0.167
Elusimicrobia	0.0014	0.0022	0.980
Chloroflexi	0.0003	0.0017	0.289
Synergistetes	6,57E-01	7,72E-01	0.323
Chlamydiae	3,75E-01	8,72E-02	0.012

**Table 2:** Class to genus level of the sheep hindgut microbiome in the two groups (isolated, control). Relative abundances. OTU: operational taxonomic units.

Taxa	OTU	Control	Isolated
Class	Clostridia	0.4047	0.4260
Class	Bacteroidia	0.2763	0.3270
Class	Epsilonproteobacteria	0.1090	0.0709
Class	Verrucomicrobiae	0.0860	0.0628
Order	Clostridiales	0.4041	0.4254
Order	Bacteroidales	0.2763	0.3270
Order	Campylobacterales	0.1090	0.0709
Order	Verrucomicrobiales	0.0860	0.0628
Family	*Ruminococcaceae*	0.2807	0.2923
Family	Rikenellaceae	0.0994	0.1107
Family	Campylobacteraceae	0.0975	0.0610
Family	Verrucomicrobiaceae	0.0860	0.0628
Family	Prevotellaceae	0.0564	0.0804
Family	Bacteroidaceae	0.0516	0.0648
Genus	*Ruminococcaceae* UCG-010	0.0970	0.0801
Genus	Campylobacter	0.0975	0.0610
Genus	Akkermansia	0.0860	0.0628
Genus	*Ruminococcaceae* UCG-005	0.0614	0.0717
Genus	Bacteroides	0.0516	0.0648

**Table 3:** Main taxa differences in the sheep hindgut microbiome between the two different husbandry conditions. The column Bayesian 10% reports the probability that the difference between the two groups is larger than the threshold (see material and methods section), estimated from a Bayesian model.

Level	Taxon	Control	Isolated	*p*-Value	Bayesian 10%
phylum	Fibrobacteres	3545	708.2	0.0293	0.928
class	Fibrobacteria	3545	708.2	0.0293	0.939
order	Aeromonadales	28.6	6	0.0132	0.956
order	Desulfovibrionales	511.8	268.8	0.0042	0.967
order	Fibrobacterales	3545	708.2	0.0293	0.936
order	Micrococcales	167.6	72.6	0.0299	0.924
order	Thermoanaerobacterales	118.4	66.2	0.0138	0.939
family	Defluviitaleaceae	212	108.8	0.0119	0.945
family	Dermatophilaceae	161	67.4	0.0266	0.929
family	Desulfovibrionaceae	511.8	268.8	0.0042	0.967
family	Fibrobacteraceae	3545	708.2	0.0293	0.935
family	Methylobacteriaceae	2.4	0.2	0.0302	0.949
family	Succinivibrionaceae	28.6	6	0.0132	0.957
family	Thermoanaerobacteraceae	118.4	66.2	0.0138	0.938
genus	Asteroleplasma	200.8	65	0.0442	0.956
genus	Catenibacterium	81.6	20.8	0.0154	0.953
genus	Defluviitaleaceae UCG-011	212	108.8	0.0119	0.949
genus	Desulfovibrio	504.4	264	0.0046	0.966
genus	Fibrobacter	3545	708.2	0.0293	0.937
genus	Gelria	118.4	66.2	0.0138	0.938
genus	Lachnoclostridium 10	754.6	328.4	0.0045	0.971
genus	Methylobacterium	2.4	0.2	0.0302	0.948
genus	Rikenellaceae RC9 gut groupgutgroup	8569.2	6187.4	0.0366	0.877
genus	Ruminobacter	28.6	6	0.0132	0.959
genus	*Ruminococcaceae* UCG-011	1140.2	615.2	0.0025	0.975
genus	Solobacterium	1	10.8	0.0248	0.945
genus	uncultured organism unculturedorganism	67.8	24.4	0.0310	0.918

**Table 4:** Firmicutes to Bacteroides (F/B) ratio between control and isolated group and *p*-value of difference (*p*-value = 0.436).

Group	F/B_avg	B_avg	F_avg	F/B_med	B_med	F_med
Control	1.54	0.28	0.42	1.50	0.29	0.43
Isolated	1.40	0.33	0.44	1.30	0.32	0.45

**Table 5:** Average alpha diversity indices (both richness and evenness) for the sheep hindgut microbiome. All samples irrespective of husbandry condition. ACE: abundance-based coverage estimator.

Index.	*n*	Avg_v	Std
Chao1	10	2907	112.3
ACE	10	2902	104.9
Fisher_alpha	10	448.9	20.5
Observed_otus	10	2592.7	141.6
Shannon	10	8.5	0.38
Simpson	10	0.986	11
Equitability	10	0.753	0.03
Simpson_e	10	0.041	0.002

**Table 6:** Summary of estimated alpha diversity indices in the sheep hindgut microbiome from the two experimental groups. *p*-values for among-group differences from analysis of variance (*p*-value) and Bayesian results (Bayesian probability 10%). The column Bayesian 10% reports the probability, estimated from a Bayesian model, that the difference between the two groups is larger than the threshold (see M&M).

Alpha Diversity	Control (*n* = 5)	Isolated (*n* = 5)	*p*-Value	Bayesian Prob. 10%
Chao1	2955.2, +/−104.29	2860.5, +/−109.2	0.198	0.044
ACE	2938.6, +/−108.6	2867.237, +/−99	0.309	0.032
Fisher_alpha	442.27, +/−20.31	455.72, +/−20.54	0.328	0.069
Observed_otus	2648+/−129.3	2537.4 +/−144.1	0.237	0.131
Shannon	8.4, +/−0.43	8.7, +/−0.26	0.203	0.072
Simpson	0.98, +/−0.015	0.99, +/−0.003	0.178	0.001
Equitability	0.74 +/−0.04	0.77 +/−0.019	0.151	0.084
Simpson_e	0.03, +/−0.021	0.05 +/−0.013	0.177	0.776
